# Association Between Physical Activity and Pelvic Floor Disorders in Parous Ugandan Women

**DOI:** 10.1007/s00192-024-05859-4

**Published:** 2024-07-13

**Authors:** Julia Diane Fleecs, Michael Derrick Ngobi, Flavia Matovu Kiweewa, Ramya Vemulapalli, JaNiese Elizabeth Jensen, Haley Alaine Steffen, Linder Hagstrom Wendt, Jay Brooks Jackson, Kimberly Ann Kenne

**Affiliations:** 1 University of Iowa Carver College of Medicine, Iowa City, IA USA; 2 Makerere University-John Hopkins University Research Collaboration, Kampala, Uganda; 3 University of Iowa Institute for Clinical and Translational Science, Iowa City, IA USA; 4 Department of Pathology, Carver College of Medicine, University of Iowa, Iowa City, IA USA; 5 Department of Obstetrics and Gynecology, University of Iowa, 200 Hawkins Drive, Iowa City, IA 31674 PFP USA

**Keywords:** Pelvic floor disorders, Physical activity, Parous women, Ugandan women

## Abstract

**Introduction and Hypothesis:**

The aim was to assess the association between the degree of physical activity (PA) and the presence of pelvic floor disorders (PFDs) in a cohort of parous Ugandan women.

**Methods:**

In this cross-sectional study, PFDs were measured using symptom assessment, standardized questionnaires (Pelvic Floor Distress Inventory and Pelvic Floor Impact Questionnaire), and a standardized physical examination (POP-Q and cough stress test [CST]). Degree of PA was assessed using the International Physical Activity Questionnaire. Interquartile ranges were used to describe the age, parity, and body mass index (BMI) of participants. To examine the association between PA and PFDs, a log transformation was applied to the weekly minutes of PA variable and a logistic regression model was constructed with weekly minutes of moderate/vigorous PA, age, BMI, and parity as the predictors.

**Results:**

A total of 159 women were enrolled. Median age was 35 (IQR 32–37), median parity 4 (IQR 3–5), and median BMI 29.0 (IQR 24–33). The prevalence of PFD as determined by symptom assessment was 28% (n=44). The most frequent stage of prolapse identified by POP-Q was stage II (57%, *n*=91). Thirty-six percent of the women (*n*=58) reported vigorous PA. Ninety-nine percent of the cohort (*n*=158) reported moderate PA. When controlling for age, parity, and BMI there was a significant positive association between PFD (defined as a combination of stage II prolapse, positive CST, and urinary incontinence (UI)) and moderate PA (OR 2.20, 95% CI 1.08–5.14, *p* value 0.045).

**Conclusions:**

Pelvic floor disorders are common among parous Ugandan women and are associated with moderate PA when controlling for age, BMI, and parity. Understanding the risk factors associated with PFD in this population may better equip providers to screen and care for individuals.

## Introduction

Pelvic floor disorders (PFDs) comprise a set of conditions including urinary incontinence (UI), anal incontinence (AI), and pelvic organ prolapse (POP). Prolapse is characterized by loss of vaginal support with descent of the uterus, bladder, and or rectum resulting in pelvic pain, difficulty with emptying the bladder and or bowel, and urinary and fecal incontinence [1].

Pelvic floor disorders are multifactorial conditions with known risk factors of higher parity, younger age at first delivery, poor nutrition, obesity, and less access to obstetric and gynecological care [1]. These disorders are a significant women’s health issue, affecting quality of life for women worldwide. In the USA, approximately 25% of women suffer from at least one PFD [2]. A more recent study from 2022 found that 32% of women seen in a primary care setting in the USA had at least one PFD, with the most common being AI [3]. Unfortunately, this assessment of prevalence and need for intervention cannot be made in most low- and middle-income countries (LMICs). A review of the prevalence of PFD in LMICs had a pooled prevalence of 25%, although the authors mentioned a need for population-based research for more reliable estimates [4]. Data on PFDs in LMICs remain scarce, despite these women having more pregnancies, poorer nutrition, and less access to obstetric and gynecological care than women in higher income countries (HICs), potentially increasing the risk for PFD and more profoundly impacting quality of life [1, 5].

The relationship of physical activity (PA) to PFDs has been debated in the literature and identified as a potentially modifiable risk factor. A review of studies carried out in the USA found that UI during exercise is common and more prevalent in women during high-impact sports, although mild to moderate PA, such as brisk walking, decreases the odds of having and the risk of developing UI [6]. The same review found that women undergoing surgery for POP were more likely to report a history of heavy work than controls; however, women recruited from the community with POP on examination report similar lifetime levels of strenuous activity to women without the examination finding [6]. A Ugandan study reports that PA that increases intra-abdominal pressure, such as farming, is a risk factor for POP [7]. Data remain insufficient to determine whether strenuous activity predisposes individuals to PFD.

In LMICs, women are likely to have increased PA during transportation, occupation, and daily household chores, potentially predisposing to higher rates of PFD. Although the degree to which PA can be modified in one’s life may be different in LMICs than HICs, it remains crucial to understand its impact on PFDs. Understanding this factor may help to identify women at a higher risk and provide the appropriate screening and intervention.

To improve access to quality-of-life treatment in Uganda, a knowledge of the prevalence and risk factors specific to Ugandan women is necessary. The aim of this cross-sectional study is to examine the association between the degree of PA and the presence of PFD in a cohort of parous Ugandan women. We hypothesize that increased PA is associated with an increased presence of PFD.

## Materials and Methods

This study was a cross-sectional study conducted in Uganda. Participants enrolled in a NIH-funded study (Maternal Bone Health Study) were offered co-enrollment in the PFD study from June 2022 to June 2023. Inclusion criteria for the Maternal Bone Health study were women who were willing and able to provide informed consent, aged 18 years and above, with a documented HIV test result, who lived in the study catchment area with no plans to move, and who agreed to not participate in other research studies involving drugs or medical devices during the study period. Additional inclusion criteria for the PFD study included being parous and willing and able to have a pelvic examination. All women regardless of PA level (active or non-active) were included in the study. Exclusion criteria were recent treatment for PFDs and inability or unwillingness to complete a pelvic examination. Institutional Review Board (IRB) approvals were obtained through the appropriate boards. After consent, a counselor administered questionnaires in Luganda, the native language of study participants. Descriptions and explanations were provided to participants regarding PFDs, including the use of images to aid understanding. Data were entered into a secure online database, Research Electric Data Capture (REDCap).

Enrollment and consent were completed during scheduled appointments. The presence of PFDs was measured in three ways: a symptom assessment, standardized questionnaires (Pelvic Floor Distress Inventory [PFDI-20], and Pelvic Floor Impact Questionnaire [PFIQ-7]), and a standardized physical examination (pelvic organ prolapse quantification [POP-Q] examination and a cough stress test [CST]). Symptom assessment included an interview regarding the presence of UI, POP, and AI, as well as self-reported frequency (less than once a month, more than once a month, more than once a week, more than once a day). The PFDI-20 provided an evaluation of both the presence or absence of disorders and assessment of the degree of bother from that disorder using 20 questions across three scales (Urinary Distress Inventory, Pelvic Organ Prolapse Distress Inventory, and Colorectal-Anal Distress Inventory). Degree of bother was ranked from 0 to 4, 0 being “not present” and 4 being “a lot” [8]. The PFIQ-7 used seven questions to assess how the presence of PFD impacts a woman’s life in relation to daily activities, relationships, and feelings [8]. POP-Q staged prolapse from stage 0 to Stage 4, with no prolapse observed to total vaginal eversion, respectively. Measurements rely on the position of six distinct locations measured during a maximum Valsalva with the hymen as a reference point [9].

Socio-demographic data and clinical variables were obtained from participant interviews and participant medical records. Variables included age, parity, and body mass index (BMI). Prior to data collection and examination, training was provided to study personnel on physical examination and interview techniques that ensured accuracy and sensitivity to participants’ culture and values.

The degree of PA was assessed using the International Physical Activity Questionnaire (IPAQ) [8], which highlighted activity related to occupation, travel, and leisure. PA related to occupation/work was defined as either moderate-intensity or vigorous-intensity. “Vigorous-intensity activities” were those that required hard physical effort and caused large increases in breathing or heart rate. Examples given included heavy lifting, digging, carrying heavy loads, cutting and fetching firewood, collecting water for household use, farming activities, and fast bicycling for at least 10 min consecutively. “Moderate-intensity activities” were activities that required moderate physical effort and caused small increases in breathing or heart rate. Examples given included brisk walking, carrying light loads, bicycling at a regular pace, household chores such as sweeping, mopping, and polishing floors for at least 10 min consecutively. Recreational activities such as sports and fitness were also assessed, as was sedentary behavior. Results were reported in minutes/week.

Continuous variables were summarized using medians and inter-quartile ranges (IQRs), whereas categorical variables were summarized using counts and percentages. To examine the association between minutes of exercise and PFDs, a log transformation was applied to the weekly minutes of exercise variable and regression models were constructed with weekly minutes of moderate or vigorous exercise, age, BMI, and parity as the predictors. Age, BMI, and parity were chosen as controls for confounders as they are known risk factors for PFDs [3]. Right-skewed continuous outcomes were modeled using gamma regression with a log link, whereas binary outcomes were modeled using logistic regression. *p* values less than 0.05 were considered statistically significant and R version 4.3.1 was used for all analyses.

## Results

A total of 163 individuals in the Maternal Bone Health Study were screened; 4 individuals were not interested in the PFD study, leaving 159 women enrolled with a median age of 35 (IQR 32–37), median age at first delivery of 19 (IQR 17–21), median parity of 4 (IQR 3–5), and median BMI of 29.0 (IQR 24.3–33.3). Additional demographic data are summarized in Table 1.

**Table 1:** Demographic data

Demographic factor	*N*=159
Age at first delivery, median (IQR)	19 (17–21)
Age	35 (32–37)
Parity	4 (3–5)
Gravity	4 (3–6)
Body mass index	29.0 (24.3–33.3)
Mode of delivery, *n* (%)	
Only vaginal deliveries	91 (57)
Only cesarean deliveries	21 (13)
Both vaginal and cesarean deliveries	47 (30)
Occupation, *n* (%)	
Farmer	16 (10)
Homemaker	2 (1.3)
Cook	17 (11)
Manual laborer	1 (0.6)
Merchant	48 (30)
Unemployed	33 (21)
Other	42 (26)
Location of deliveries, *n* (%)	
Only home deliveries	1 (0.6)
Only hospital deliveries	135 (85)
Both home and hospital deliveries	23 (14)
Instrumented deliveries, *n* (%)	
Neither	153 (96)
Vacuum	5 (3.1)
Forceps	1 (0.6)
Laceration at time of delivery, *n* (%)	
Unknown	21 (13)
None	57 (36)
1st degree	34 (21)
2nd degree	45 (28)
3rd degree	2 (1.3)
4th degree	0 (0)

*IQR* interquartile range

The prevalence of PFDs as determined by symptom assessment was 28% (*n*=44). The most frequent stage of prolapse as determined by POP-Q was stage II (57%, *n*=91). 72 women (46%) had a positive CST, with a median bladder volume of 150 ml. Additional information regarding prevalence of PFDs in this cohort is summarized in Tables 2 and 3.

**Table 2:** Prevalence of pelvic floor disorders by participant report

Participant-reported symptoms	*N*=159
Urinary incontinence, *n* (%)	44 (28)
If yes: frequency	
Less than once a month	16 (36)
More than once a month	20 (45)
More than once a week	7 (16)
More than once a day	1 (2.3)
Anal incontinence	0 (0)
Pelvic organ prolapse	0 (0)
PFDI-20, median (IQR)	
Summary score	17 (0–31)
POPDI	0 (0–8)
CRADI-8	0 (0–13)
UDI-6	0 (0–17)
PFIQ-7, median (IQR)	
Summary score	0 (0–0)
UIQ7	0 (0–0)
CRAIQ7	0 (0–0)
POPIQ7	0 (0–0)

*IQR* interquartile range, *PFDI* Pelvic Floor Distress Inventory, *POPDI* Pelvic Organ Prolapse Distress Inventory, *CRADI* Colorectal-Anal Distress Inventory, UDI Urinary Distress Inventory, *PFIQ* Pelvic Floor Impact Questionnaire, *UIQ* Urinary Impact Questionnaire, *CRAIQ* Colo-Rectal-Anal Impact Questionnaire, *POPIQ* Pelvic Organ Prolapse Impact Questionnaire

**Table 3:** Prevalence of pelvic floor disorders as defined by physical examination

Physical examination characteristics	*N*=159
POP-Q stage, *n* (%)	
Stage 1	66 (42)
Stage 2	91 (57)
Stage 3	2 (1.3)
Prolapse to or beyond the hymen	20 (13)
Cough stress test, *n* (%)	
Positive	72 (46)
Negative	86 (54)

A total of 58 women (36%) reported vigorous PA and the overall median level of 0 (IQR 0–210) minutes/week. A total of 158 women (99%) reported moderate PA, with a median level of 1,260 (IQR 840–2,100) minutes/week. All women who reported vigorous PA also reported moderate PA (Table 4).

**Table 4:** Summary of physical activity (*PA*)

Level of PA	*N* (%), (minutes per week, IQR)
Vigorous PA (total), *n* (%), (minutes per week, IQR)	58 (36), 0 (0–210)
Work	54 (34)
Leisure	6 (3.8)
Moderate PA (total), *n* (%), (minutes per week, IQR)	158 (99), 1,260 (840–2,100)
Work	158 (99)
Leisure	32 (20)

When controlling for age, parity, and BMI, there were very few significant positive associations between the presence of PFDs, regardless of definition (symptom assessment, standard questionnaires, or standard physical examination) and level of PA as defined as minutes per week of either vigorous or moderate PA in this cohort of parous Ugandan women. However, we did observe that greater levels of moderate PA corresponded with a significantly increased risk of PFD when PFD is defined as the combination of stage II or greater prolapse, a positive CST, and UI (mean ratio 2.20, 95% CI 1.08–5.14, *p* value 0.045; Table 5). Additionally, we observed that vigorous PA had a likely negative relationship with PFDI-20 scores, but this was only marginally significant (mean ratio 0.97, 95% CI 0.93–1.01, *p*=0.087).

**Table 5:** Presence of pelvic floor disorder (*PFD*) by level of physical activity (*PA*) (min/week) when controlling for age at first delivery, BMI, and parity

Definition of PFD	Vigorous PA	Moderate PA
Symptom assessment: UI^a^	1.03, 0.94–1.11, 0.6	1.16, 0.86–1.68, 0.4
PFDI-20 score^b^	**0.97, 0.93–1.01, 0.087**	0.98, 0.87–1.07, 0.7
PFIQ-7 score^b^	1.03, 0.98–1.09, 0.2	0.87, 0.71–1.07, 0.2
Stage II POP or greater^a^	1.04, 0.96–1.12, 0.4	0.85, 0.62–1.12, 0.3
Positive CST^a^	0.97, 0.90–1.05, 0.5	0.95, 0.73–1.24, 0.7
Combined (UI, ≥ stage II POP, positive CST)^1^	1.08, 0.94–1.24, 0.3	**2.20, 1.08–5.14, 0.045**

*UI* urinary incontinence, *PFDI* Pelvic Floor Distress Inventory, *PFIQ* Pelvic Floor Impact Questionnaire, *POP* pelvic organ prolapse, *CST* cough stress test

^a^Results shown as OR, 95% CI, *p* value

^b^Results shown as mean ratio, 95% CI, *p* value

## Discussion

In this cohort of parous Ugandan women, the prevalence of PFD as determined by symptom assessment was 28%. This finding compares with the prevalence of symptomatic PFD reported in other LMICs (25%) [4]. It is also similar to the prevalence of symptomatic PFD found by Nygaard et al. in women in the USA (25%) [2]. A slightly higher prevalence was reported by Kenne et. al. in the primary care setting in the USA (32%) as well as in a South-Central Ethiopian study (41.1%) [3, 10].

The most frequent stage of prolapse in our cohort as determined by POP-Q was stage II (57%). This finding is consistent with a study carried out in Southwestern Uganda that also found stage II to be the most prevalent stage (63.4%), although the Southwestern Uganda study also found a higher prevalence of stage III (16.1%) and stage IV (8.9%) prolapse [7]. The women in the Southwestern Uganda study with POP were between 34 and 49 years of age. The women in our study were younger, ranging between 25 and 43 years old, potentially contributing to the difference seen in prolapse stage.

When controlling for known risk factors of PFD, there were few significant associations between the presence of PFD as defined separately by either symptom assessment, standard questionnaires, or standard physical examination and level of PA. However, when PFD was defined as the combination of stage II or greater prolapse, a positive CST, and UI, there was a correlation between greater levels of moderate PA and the presence of PFD. Additionally, a marginally significant negative relationship was seen between vigorous PA and PFDI-20 scores, as shown in Table 5.

Our results correlate with the debate found in the current literature as to whether PA is a risk factor for PFD or if it has a protective component [6]. Tugume et al. describes the former by reporting that women who were peasant farmers were more likely to have POP [7]. A comparable finding is noted in a Tanzanian study, where farmers had an almost four times increased risk of severe POP development [11]. A South-Central Ethiopian study reported that the prevalence of symptomatic PFDs was significantly associated with heavy lifting defined as >10 kg [10]. Participants in this study describe themselves fetching water using 20 liter- containers, collecting firewood, and carrying heavy objects to and from the markets. The above studies reference activities such as farming to predispose to PFD through increased intra-abdominal pressure (IAP), stretching the pelvic diaphragm [7]. This differs from our results as these activities of farming and heavy lifting would be defined as “vigorous” by the IPAQ, although our study found moderate PA to still have an association with increased risk for PFD. Interestingly, vigorous activity showed a potential for a protective relationship for PFD when considering PFDI-20 scores alone. Other studies suggest a potential protective influence of PA on PFD development, with the stipulation that the level of activity must be in just the right dose. For example, when looking at UI alone, a review found that mild to moderate PA, such as walking, decreased the risk of urinary incontinence, but female athletes who participate in more vigorous, regular exercise were almost three times more likely to have UI than controls [12]. Our findings were opposite this trend, finding moderate PA to correlate with UI, a positive CST, and stage II prolapse, whereas vigorous PA had a potentially protective relationship with PFD, as defined by PFDI-20 scores.

A review by Nygaard et al. concludes that current research suggests that most PA might not harm the pelvic floor and does provide other health benefits to women, including decreased weight gain, which is itself a risk factor for UI [6]. Our findings hint at this with a marginally significant protective relationship between vigorous PA and lower PFDI-20 scores, insinuating decreased presence and a decreased degree of bother.

The difficulty with defining a strong correlation between PA and PFD emphasizes the need for consistent quantification of the degree of PA across studies. Along with the fact that PA is often self-reported, the description as “vigorous” or “moderate” activity in one report may differ from that referenced in another. Furthermore, if under the impression that intra-abdominal pressure is the mechanism behind the impact of PA on PFD, Bø et. al. challenges that what may be considered more vigorous exercise, such as lifting 20 lbs or doing abdominal crunches, has been found to actually produce a lower mean maximal IAP than what might be considered a less vigorous activity, such as standing from a chair [12]. IAP varies widely from person to person, even when the same exercise is being performed, therefore the threshold for optimal or negative effects would be unique to an individual [12]. A more clear mechanism behind the role of PA on the pelvic floor muscles must be investigated to further attribute risk.

Some studies suggest that PA might not have a significant impact on pelvic floor muscles at all. A cross-sectional study of primiparous women 1 year post-partum who were involved in CrossFit showed no significant association between pelvic floor muscle strength (or weakness) and the measures of strength and fitness (self-reported PA, grip strength, endurance duration, etc.) [13]. Another study found that lifetime PA does not increase the odds of anatomical POP [14].

Not only do knowledge gaps prevent strong conclusions from being drawn on the relationship of PA on PFD in general, but much of the research around PA and PFD focuses on sport and intentional exercise in HICs compared with the assessment of daily physical labor women are exposed to in LMICs. Identifying the “Goldilocks” or perfect balance of PA that benefits the pelvic floor requires future prospective research, especially in countries with unique physical demands in daily life.

The strengths of this study include the use of standardized measurements for the assessment of PFD (both symptom report and standardized physical examination) and PA. The cohort of women were well characterized and unlikely to be lost to follow-up owing to the pre-existing relationship with the research collaborative. The study personnel were trained specifically for the study and had much prior experience in and knowledge of clinical trial work.

Limitations to our study include a selection bias and lack of generalizability to the entire country of Uganda. Our participants were selected from a convenience sample of participants who were enrolled in a pre-existing study and were parous. This ready accessibility allowed for ease of enrollment and follow-up but limited the generalizability of our findings. Most of the women involved were young and lived within the catchment area, further decreasing generalizability to the rest of the country in both disease course and geography. Furthermore, women who had undergone treatment for PFDs were excluded from the study, potentially excluding more advanced PFD from being evaluated in the context of PA.

Another limitation may include participant hesitation in reporting symptoms owing to the social stigma surrounding PFDs. Women in our study were offered enrollment during their visits for other unrelated appointments. This meant that they were being screened for PFDs for our research purposes, whereas in other studies, such as those in HICs, women included in PFD research are often a care-seeking population. This likely increases the probability of participants being more forthcoming about symptoms but also increases the statistical prevalence of participants with more symptomatically severe PFD. Additionally, there are no validated questionnaires specific to this population and their language.

Future studies are necessary to assess a larger, more diverse cohort in both age and geographical location within Uganda. Furthermore, continued prospective research focused on populations with unique exposure to physical demands is required to understand the true impact of PA on PFDs.

## Conclusions

Pelvic floor disorders are prevalent in a cohort of parous Ugandan women. Greater levels of moderate PA were significantly associated with increased risk of PFD when PFD was defined as the combination of stage II or greater prolapse, a positive CST, and UI. Increased levels of vigorous PA were found to be potentially protective when looking at PFDI-20 scores, indicating a decreased presence of disease and degree of bother of PFDs.

## Data Availability

The data that support these findings is available upon request to the corresponding author.
